# Supplementary material for: Genome Wide Association Study Uncovers the QTLome for Osmotic Adjustment and Related Drought Adaptive Traits in Durum Wheat
Source: Genes (Basel). 2022 Feb 2;13(2):293. doi: 10.3390/genes13020293 (PMC8871942; doi:10.3390/genes13020293)
Supplement: Supplementary file 1 [file genes-13-00293-s001.zip › Supplementary material final/Supplementary material GEC_24.1.2022 2/Figure S1.pptx]

## Slide 1
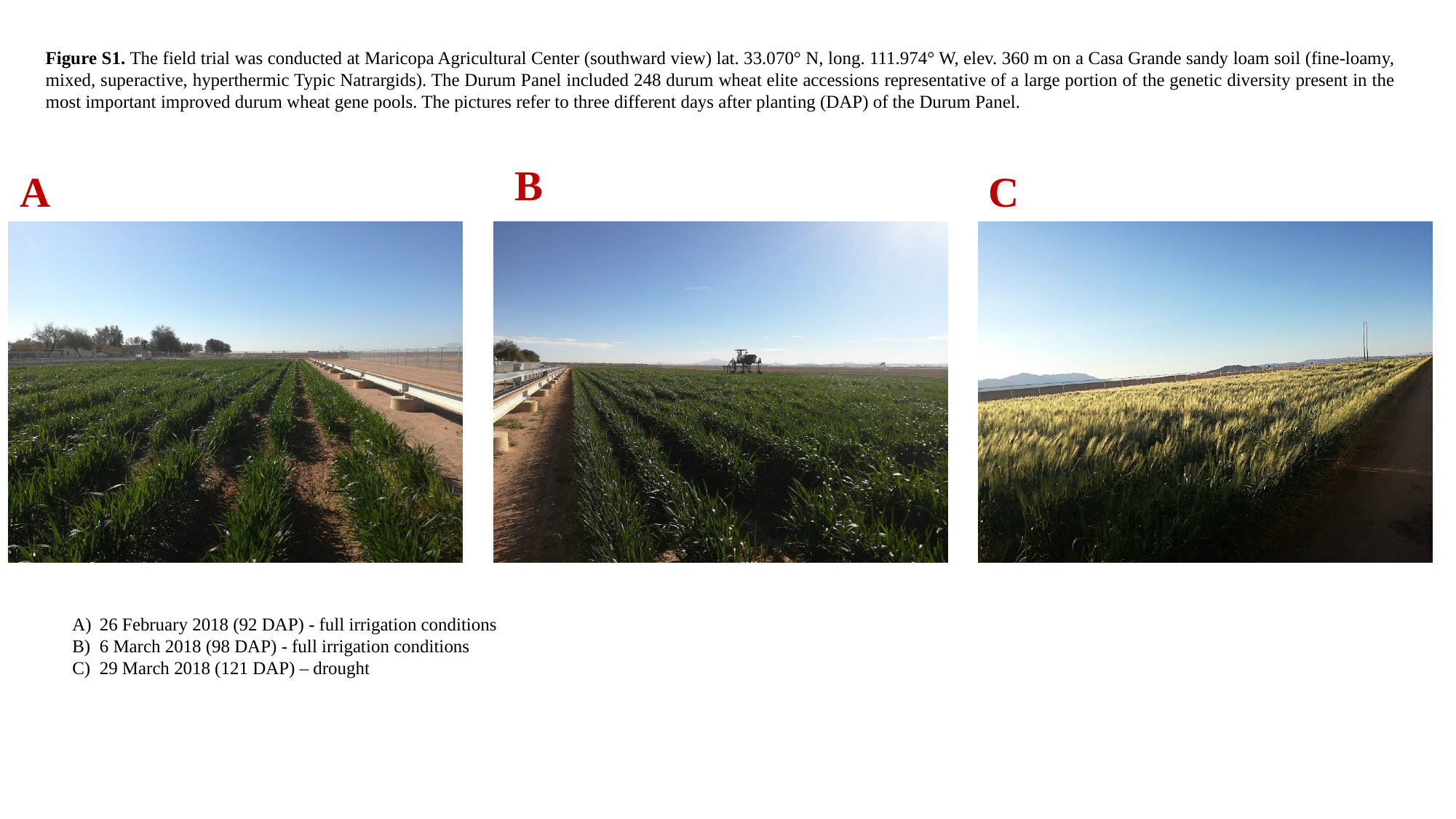

Figure S1. The field trial was conducted at Maricopa Agricultural Center (southward view) lat. 33.070° N, long. 111.974° W, elev. 360 m on a Casa Grande sandy loam soil (fine-loamy, mixed, superactive, hyperthermic Typic Natrargids). The Durum Panel included 248 durum wheat elite accessions representative of a large portion of the genetic diversity present in the most important improved durum wheat gene pools. The pictures refer to three different days after planting (DAP) of the Durum Panel.
B
C
A
26 February 2018 (92 DAP) - full irrigation conditions
6 March 2018 (98 DAP) - full irrigation conditions
29 March 2018 (121 DAP) – drought
