# Supplementary material for: Genome Wide Association Study Uncovers the QTLome for Osmotic Adjustment and Related Drought Adaptive Traits in Durum Wheat
Source: Genes (Basel). 2022 Feb 2;13(2):293. doi: 10.3390/genes13020293 (PMC8871942; doi:10.3390/genes13020293)
Supplement: Supplementary file 1 [file genes-13-00293-s001.zip › Supplementary material final/Supplementary material GEC_24.1.2022 2/Figure S2.pptx]

## Slide 1
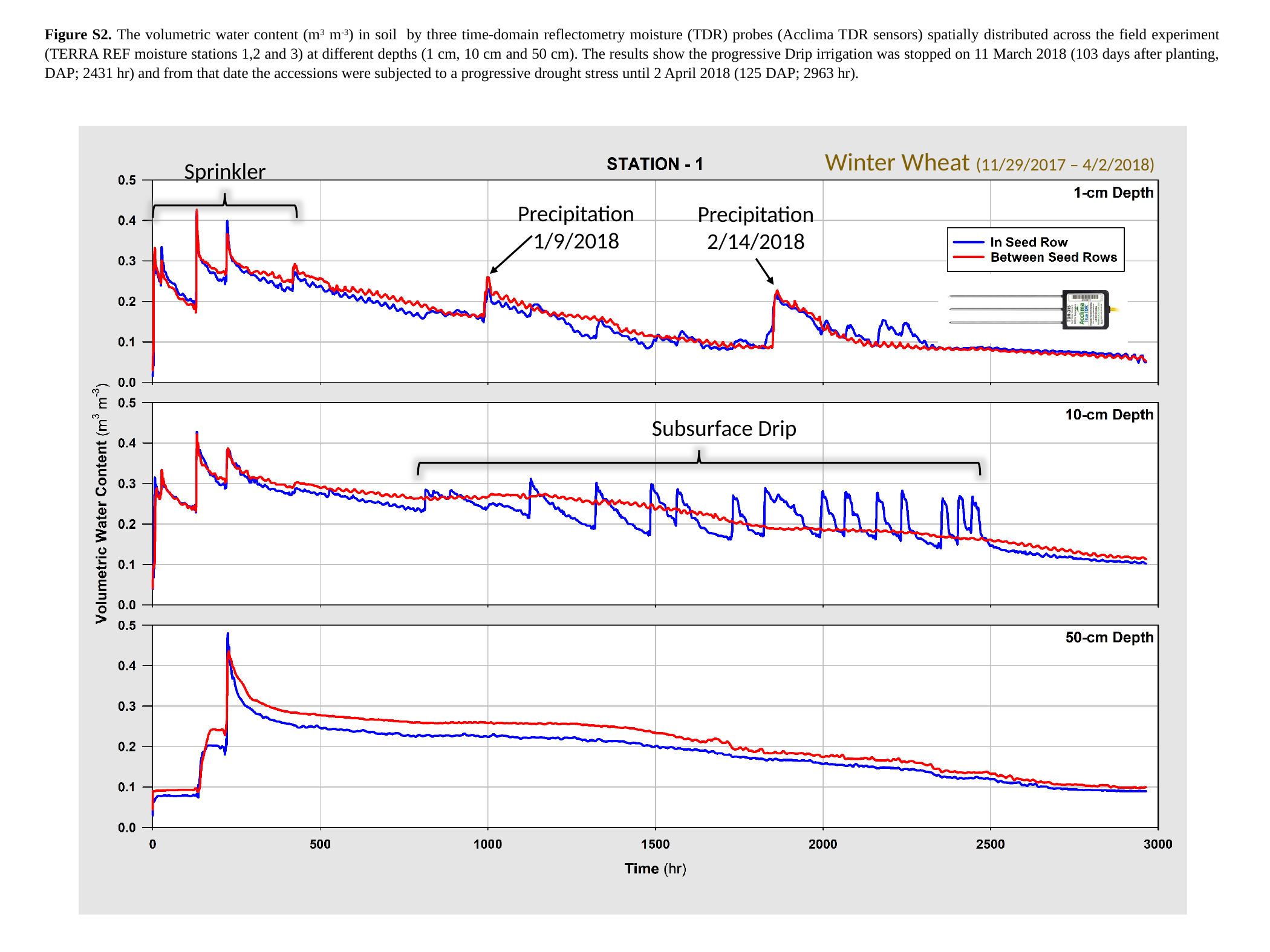

Figure S2. The volumetric water content (m3 m-3) in soil by three time-domain reflectometry moisture (TDR) probes (Acclima TDR sensors) spatially distributed across the field experiment (TERRA REF moisture stations 1,2 and 3) at different depths (1 cm, 10 cm and 50 cm). The results show the progressive Drip irrigation was stopped on 11 March 2018 (103 days after planting, DAP; 2431 hr) and from that date the accessions were subjected to a progressive drought stress until 2 April 2018 (125 DAP; 2963 hr).
Winter Wheat (11/29/2017 – 4/2/2018)
Sprinkler
Precipitation
1/9/2018
Precipitation
2/14/2018
Subsurface Drip

## Slide 2
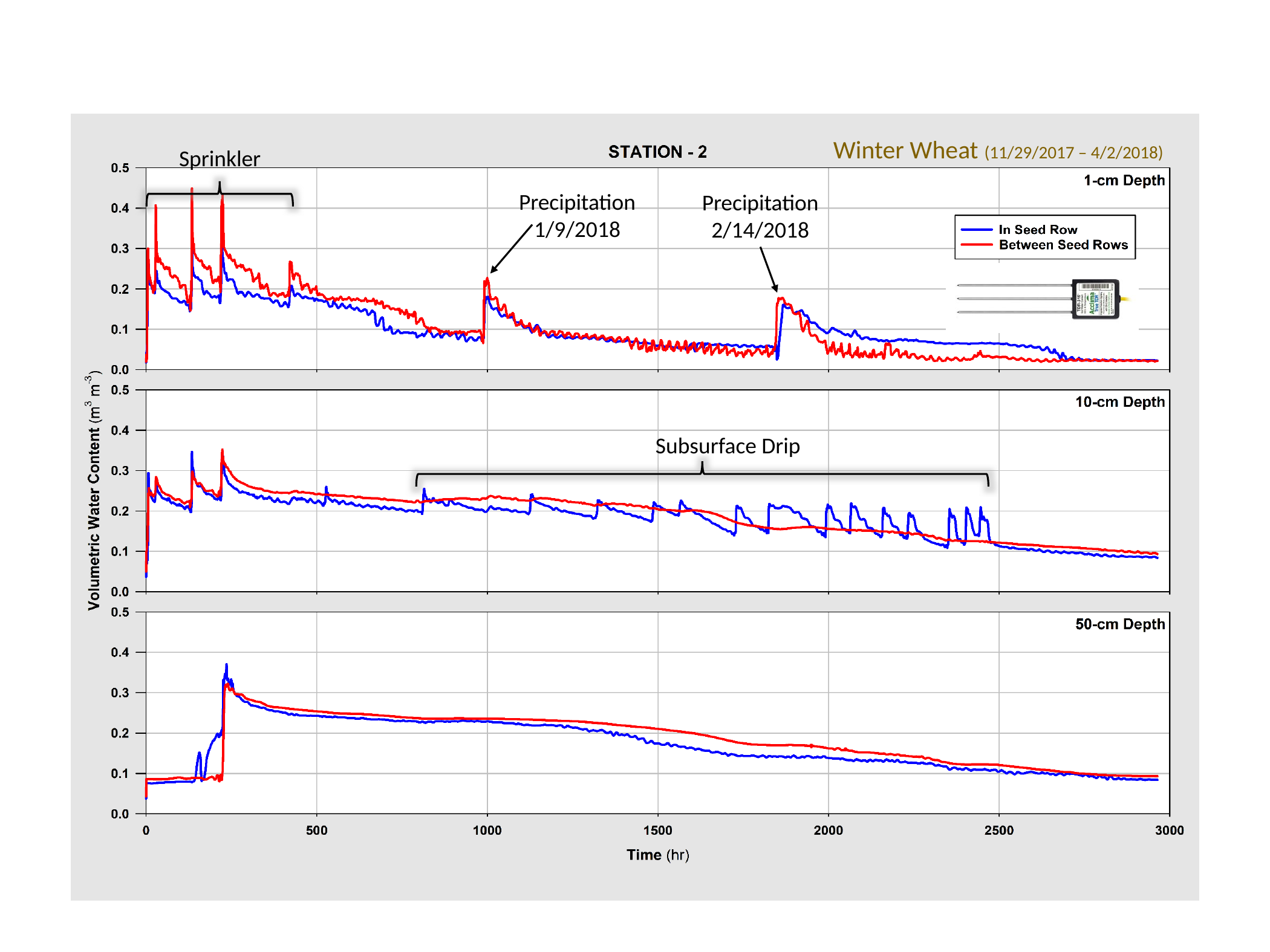

Winter Wheat (11/29/2017 – 4/2/2018)
Sprinkler
Precipitation
1/9/2018
Precipitation
2/14/2018
Subsurface Drip

## Slide 3
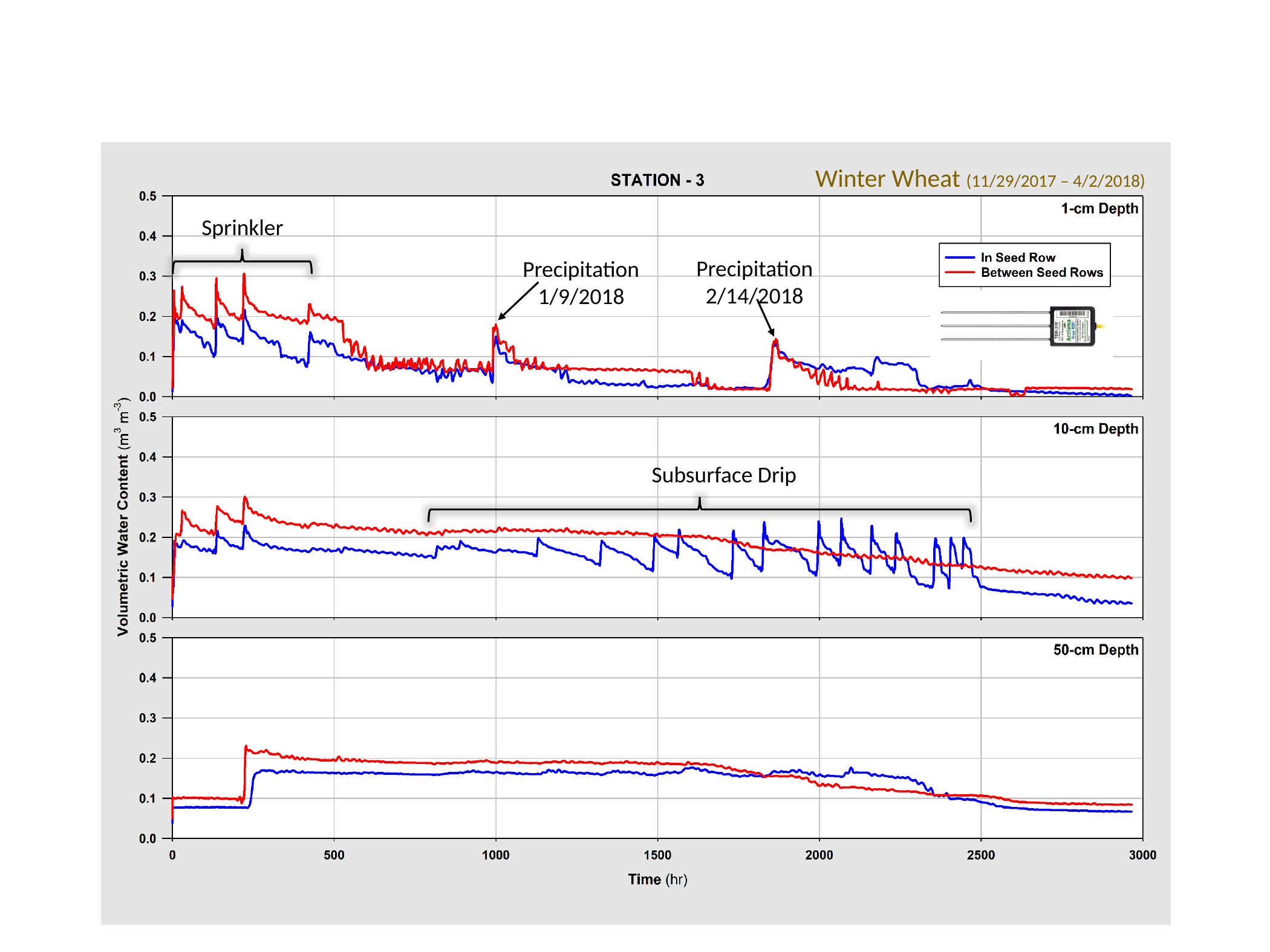

Winter Wheat (11/29/2017 – 4/2/2018)
Sprinkler
Precipitation
2/14/2018
Precipitation
1/9/2018
Subsurface Drip
