# Supplementary material for: Genome Wide Association Study Uncovers the QTLome for Osmotic Adjustment and Related Drought Adaptive Traits in Durum Wheat
Source: Genes (Basel). 2022 Feb 2;13(2):293. doi: 10.3390/genes13020293 (PMC8871942; doi:10.3390/genes13020293)
Supplement: Supplementary file 1 [file genes-13-00293-s001.zip › Supplementary material final/Supplementary material GEC_24.1.2022 2/Figure S3.pptx]

## Slide 1
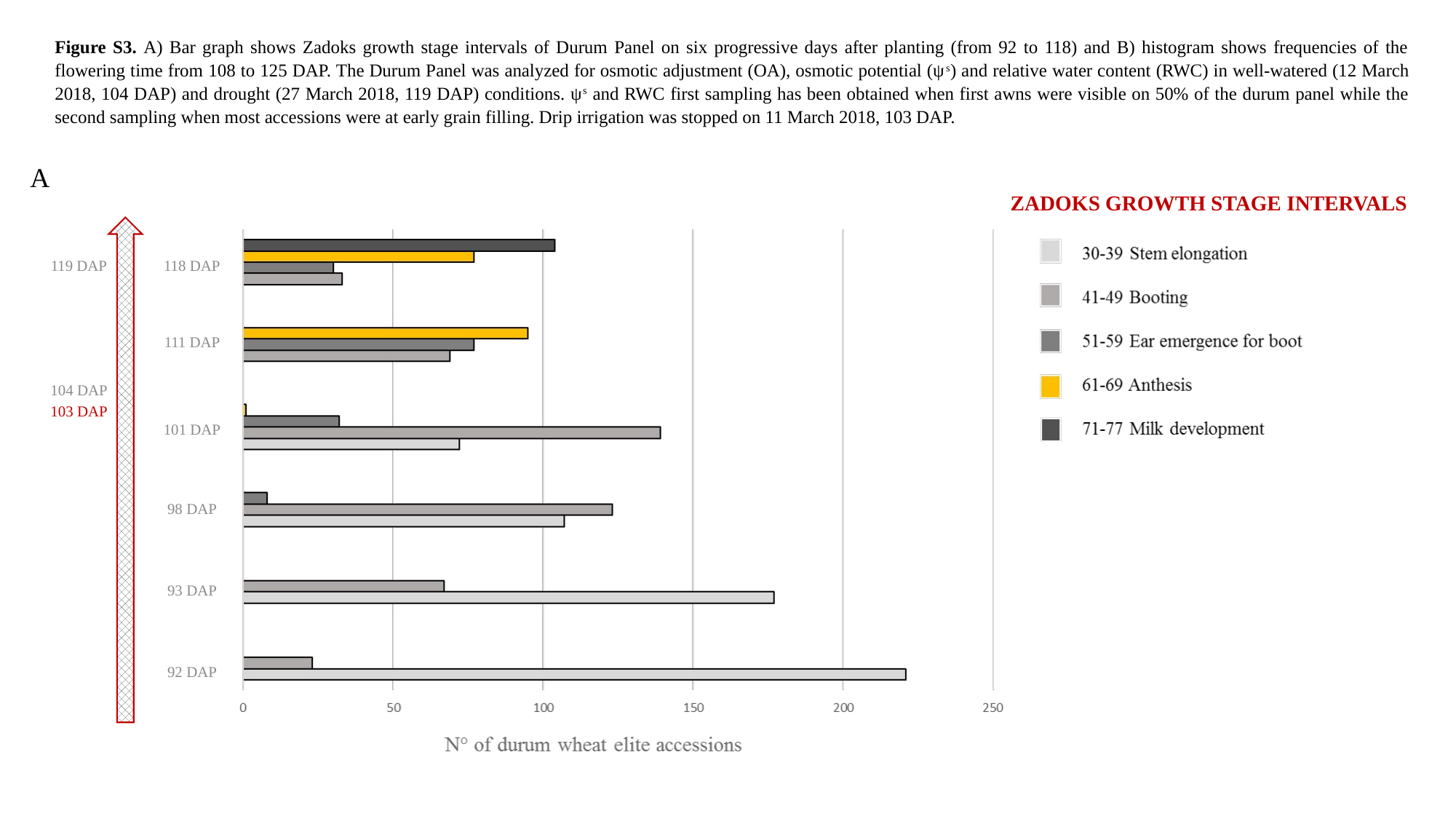

Figure S3. A) Bar graph shows Zadoks growth stage intervals of Durum Panel on six progressive days after planting (from 92 to 118) and B) histogram shows frequencies of the flowering time from 108 to 125 DAP. The Durum Panel was analyzed for osmotic adjustment (OA), osmotic potential (ψs) and relative water content (RWC) in well-watered (12 March 2018, 104 DAP) and drought (27 March 2018, 119 DAP) conditions. ψs and RWC first sampling has been obtained when first awns were visible on 50% of the durum panel while the second sampling when most accessions were at early grain filling. Drip irrigation was stopped on 11 March 2018, 103 DAP.
A
ZADOKS GROWTH STAGE INTERVALS
119 DAP
118 DAP
111 DAP
104 DAP
103 DAP
101 DAP
98 DAP
93 DAP
92 DAP

## Slide 2
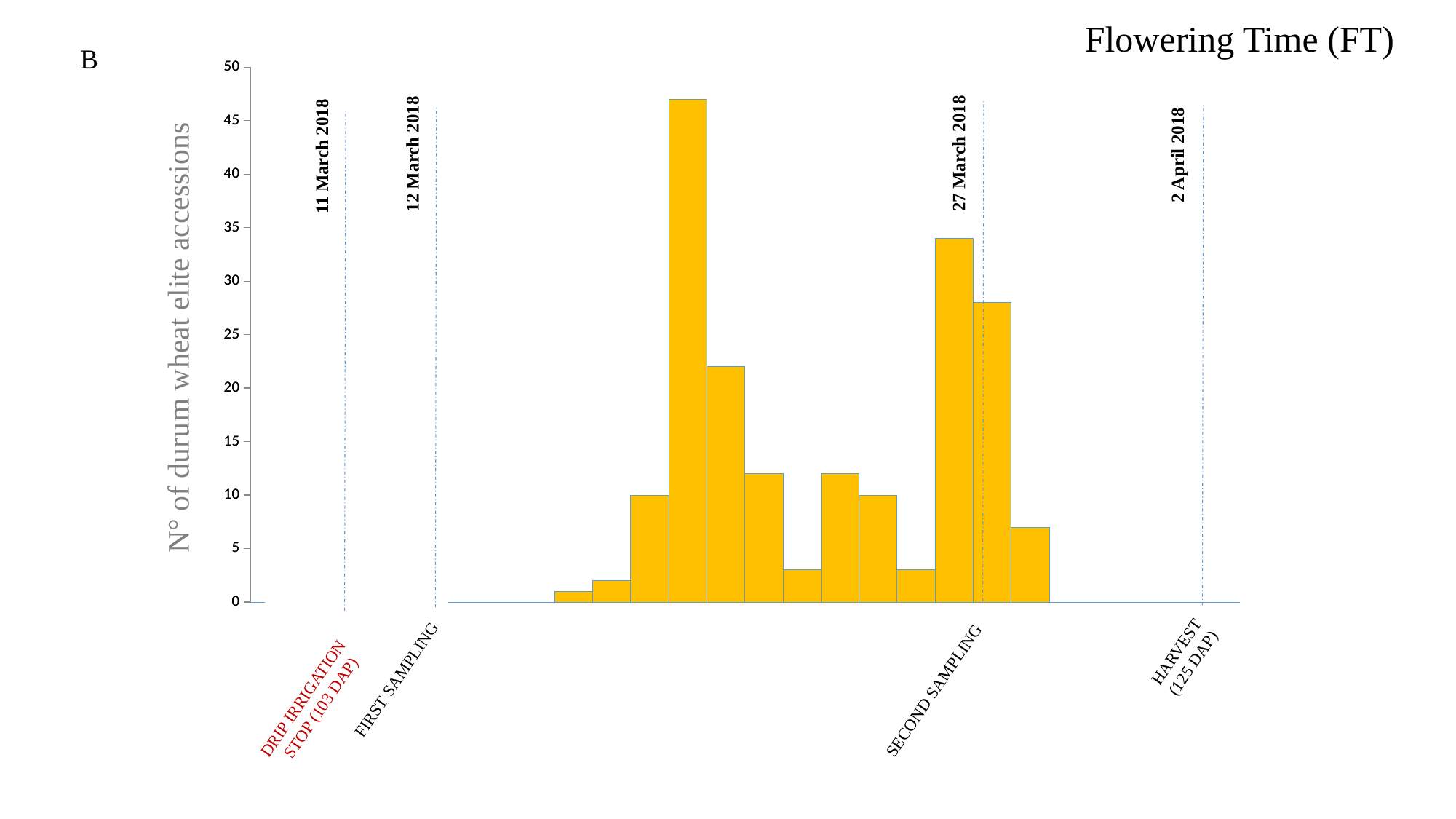

Flowering Time (FT)
B
### Chart
| Category | |
|---|---|
| 108 | 0.0 |
| 109 | 1.0 |
| 110 | 0.0 |
| 111 | 0.0 |
| 112 | 0.0 |
| 113 | 0.0 |
| 114 | 0.0 |
| 115 | 0.0 |
| 116 | 1.0 |
| 117 | 2.0 |
| 118 | 10.0 |
| 119 | 47.0 |
| 120 | 22.0 |
| 121 | 12.0 |
| 122 | 3.0 |
| 123 | 12.0 |
| 124 | 10.0 |
| 125 | 3.0 |
| 126 | 34.0 |
| 127 | 28.0 |
| 128 | 7.0 |
| 129 | 0.0 |
| 130 | 0.0 |
| 131 | 0.0 |
| 132 | 0.0 |
| 133 | 0.0 |
### Chart
| Category | |
|---|---|
| 108 | 0.0 |
| 109 | 1.0 |
| 110 | 0.0 |
| 111 | 0.0 |
| 112 | 0.0 |
| 113 | 0.0 |
| 114 | 0.0 |
| 115 | 0.0 |
| 116 | 1.0 |
| 117 | 2.0 |
| 118 | 10.0 |
| 119 | 47.0 |
| 120 | 22.0 |
| 121 | 12.0 |
| 122 | 3.0 |
| 123 | 12.0 |
| 124 | 10.0 |
| 125 | 3.0 |
| 126 | 34.0 |
| 127 | 28.0 |
| 128 | 7.0 |
| 129 | 0.0 |
| 130 | 0.0 |
| 131 | 0.0 |
| 132 | 0.0 |
| 133 | 0.0 |27 March 2018
12 March 2018
2 April 2018
11 March 2018
N° of durum wheat elite accessions
BRADANO (S6)
HARVEST
(125 DAP)
FIRST SAMPLING
SECOND SAMPLING
DRIP IRRIGATION
STOP (103 DAP)
