# Supplementary material for: Genome Wide Association Study Uncovers the QTLome for Osmotic Adjustment and Related Drought Adaptive Traits in Durum Wheat
Source: Genes (Basel). 2022 Feb 2;13(2):293. doi: 10.3390/genes13020293 (PMC8871942; doi:10.3390/genes13020293)
Supplement: Supplementary file 1 [file genes-13-00293-s001.zip › Supplementary material final/Supplementary material GEC_24.1.2022 2/Figure S5.pptx]

## Slide 1
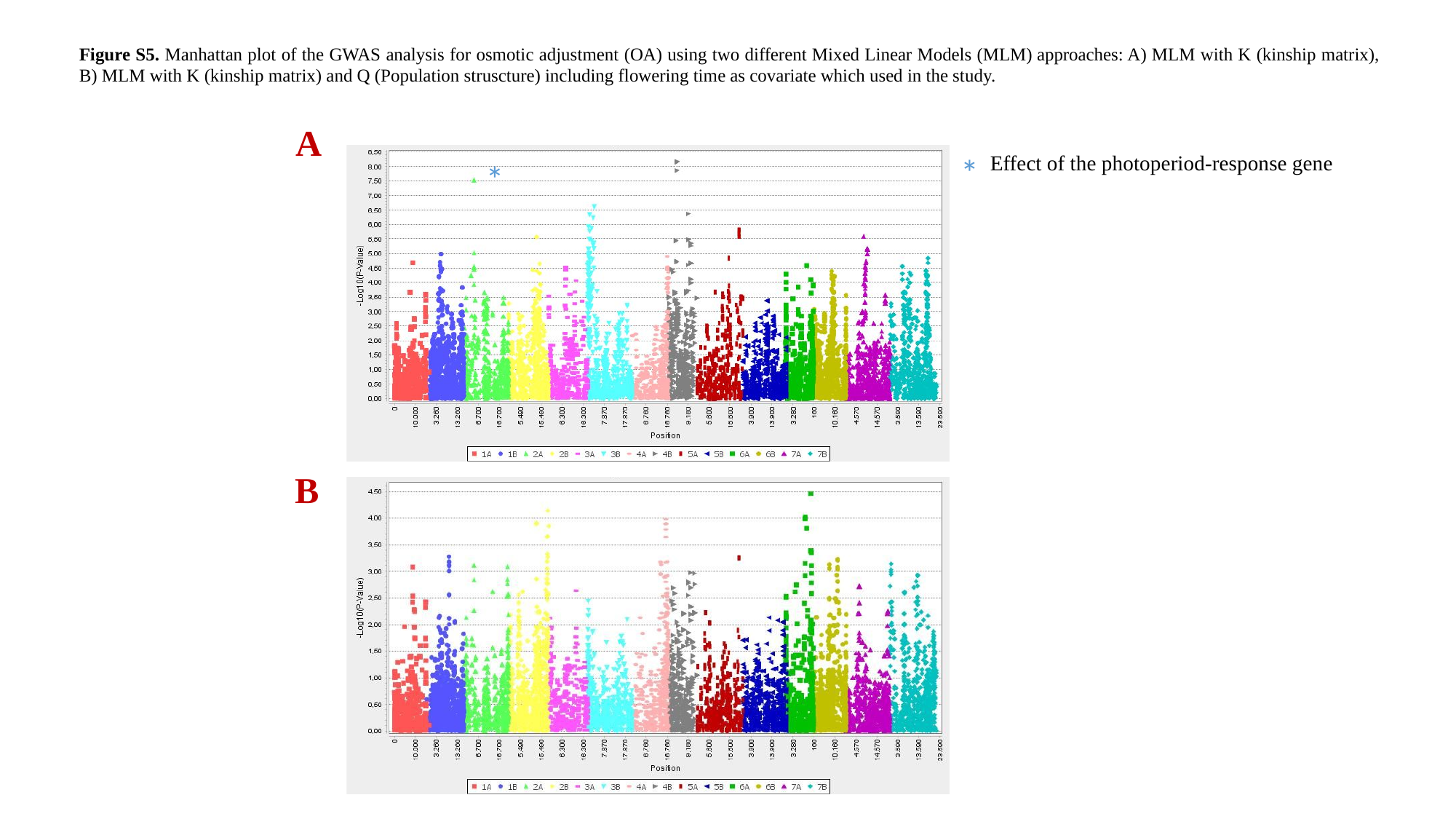

Figure S5. Manhattan plot of the GWAS analysis for osmotic adjustment (OA) using two different Mixed Linear Models (MLM) approaches: A) MLM with K (kinship matrix), B) MLM with K (kinship matrix) and Q (Population struscture) including flowering time as covariate which used in the study.
A
*
Effect of the photoperiod-response gene
*
B
