# Supplementary material for: Genome Wide Association Study Uncovers the QTLome for Osmotic Adjustment and Related Drought Adaptive Traits in Durum Wheat
Source: Genes (Basel). 2022 Feb 2;13(2):293. doi: 10.3390/genes13020293 (PMC8871942; doi:10.3390/genes13020293)
Supplement: Supplementary file 1 [file genes-13-00293-s001.zip › Supplementary material final/Supplementary material GEC_24.1.2022 2/Figure S6.pptx]

## Slide 1
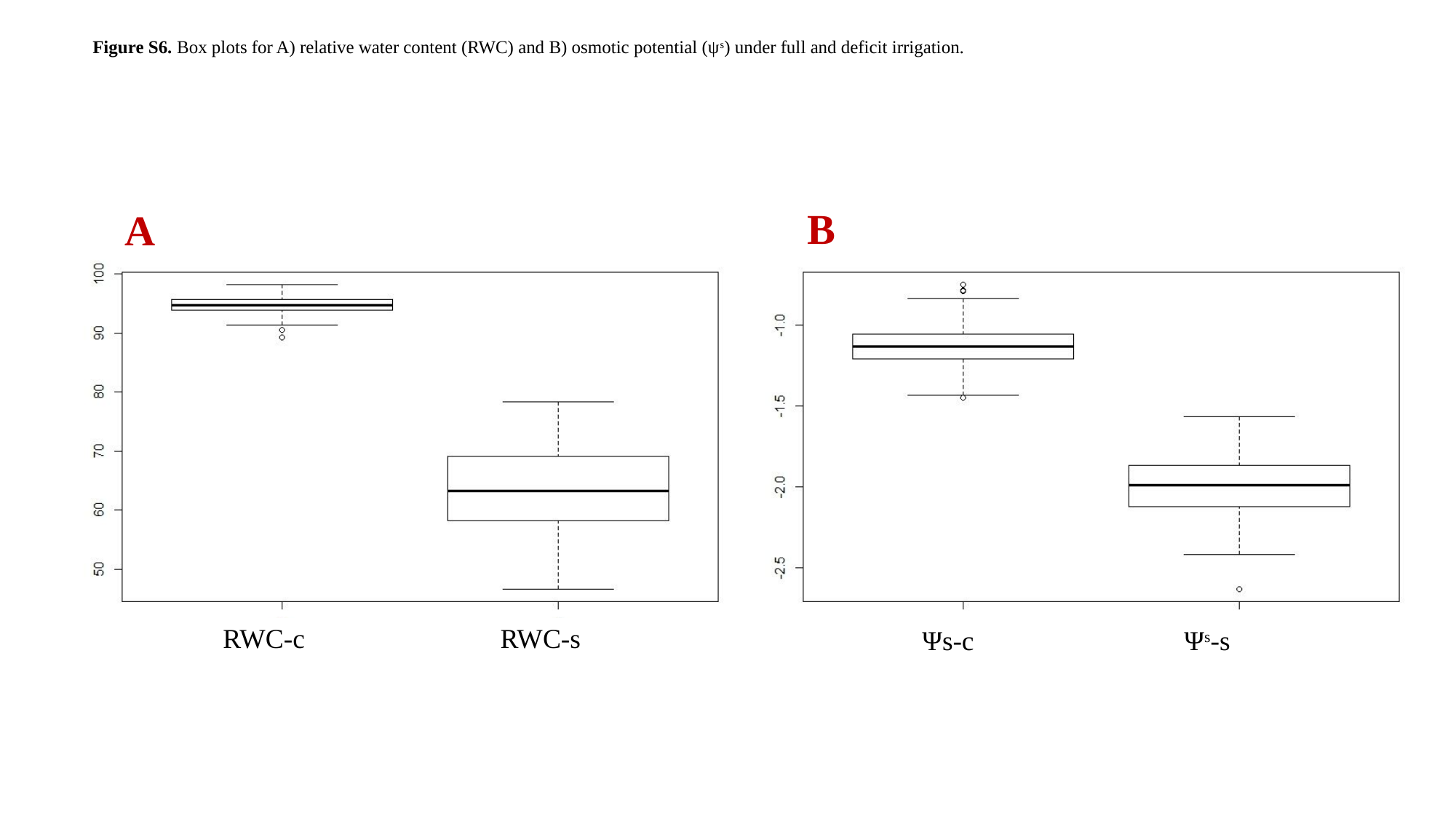

Figure S6. Box plots for A) relative water content (RWC) and B) osmotic potential (ψs) under full and deficit irrigation.
B
A
RWC-c
RWC-s
Ψs-c
Ψs-s
