# Supplementary material for: Genome Wide Association Study Uncovers the QTLome for Osmotic Adjustment and Related Drought Adaptive Traits in Durum Wheat
Source: Genes (Basel). 2022 Feb 2;13(2):293. doi: 10.3390/genes13020293 (PMC8871942; doi:10.3390/genes13020293)
Supplement: Supplementary file 1 [file genes-13-00293-s001.zip › Supplementary material final/Supplementary material GEC_24.1.2022 2/Figure S7.pptx]

## Slide 1
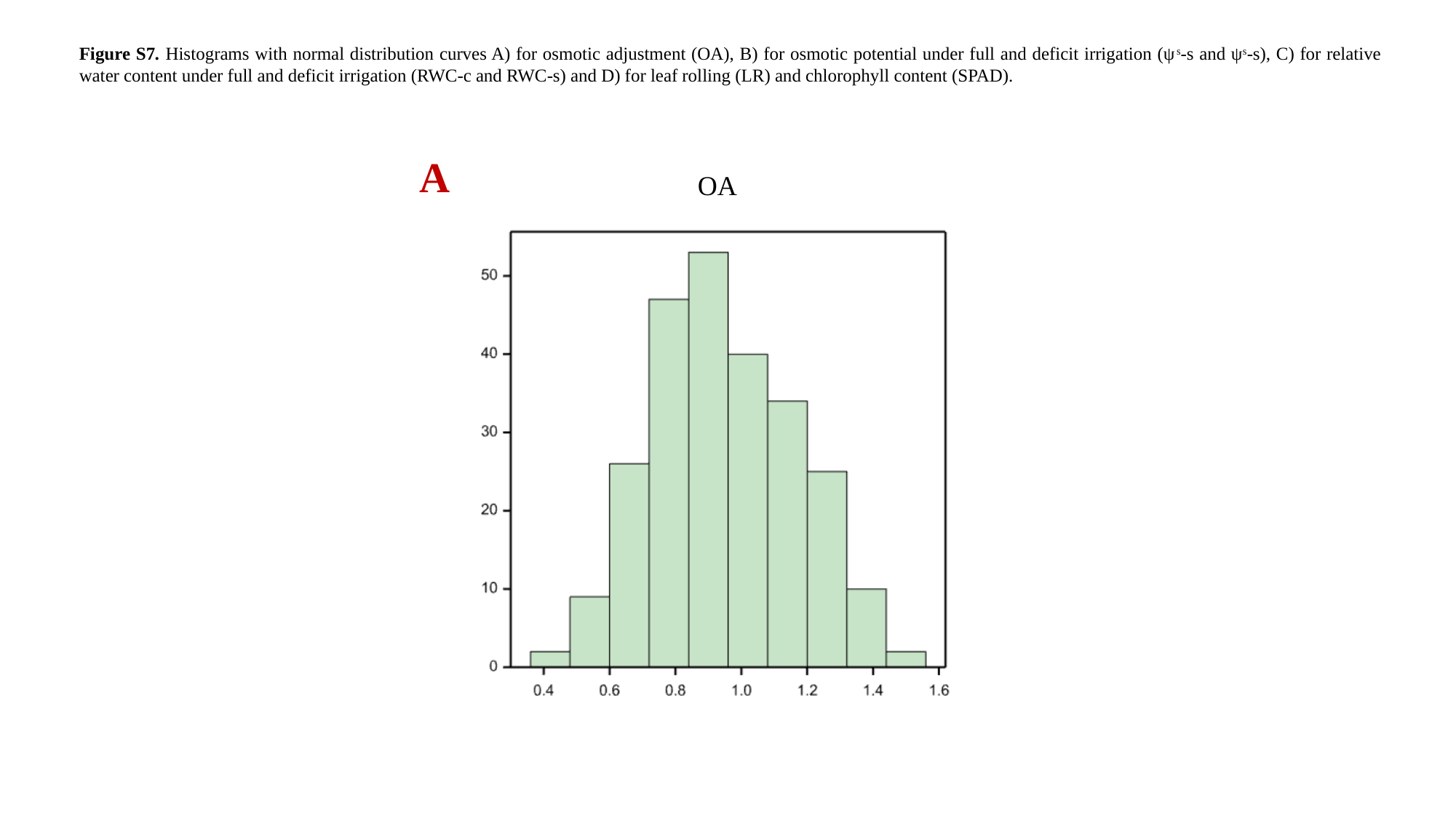

Figure S7. Histograms with normal distribution curves A) for osmotic adjustment (OA), B) for osmotic potential under full and deficit irrigation (ψs-s and ψs-s), C) for relative water content under full and deficit irrigation (RWC-c and RWC-s) and D) for leaf rolling (LR) and chlorophyll content (SPAD).
A
OA

## Slide 2
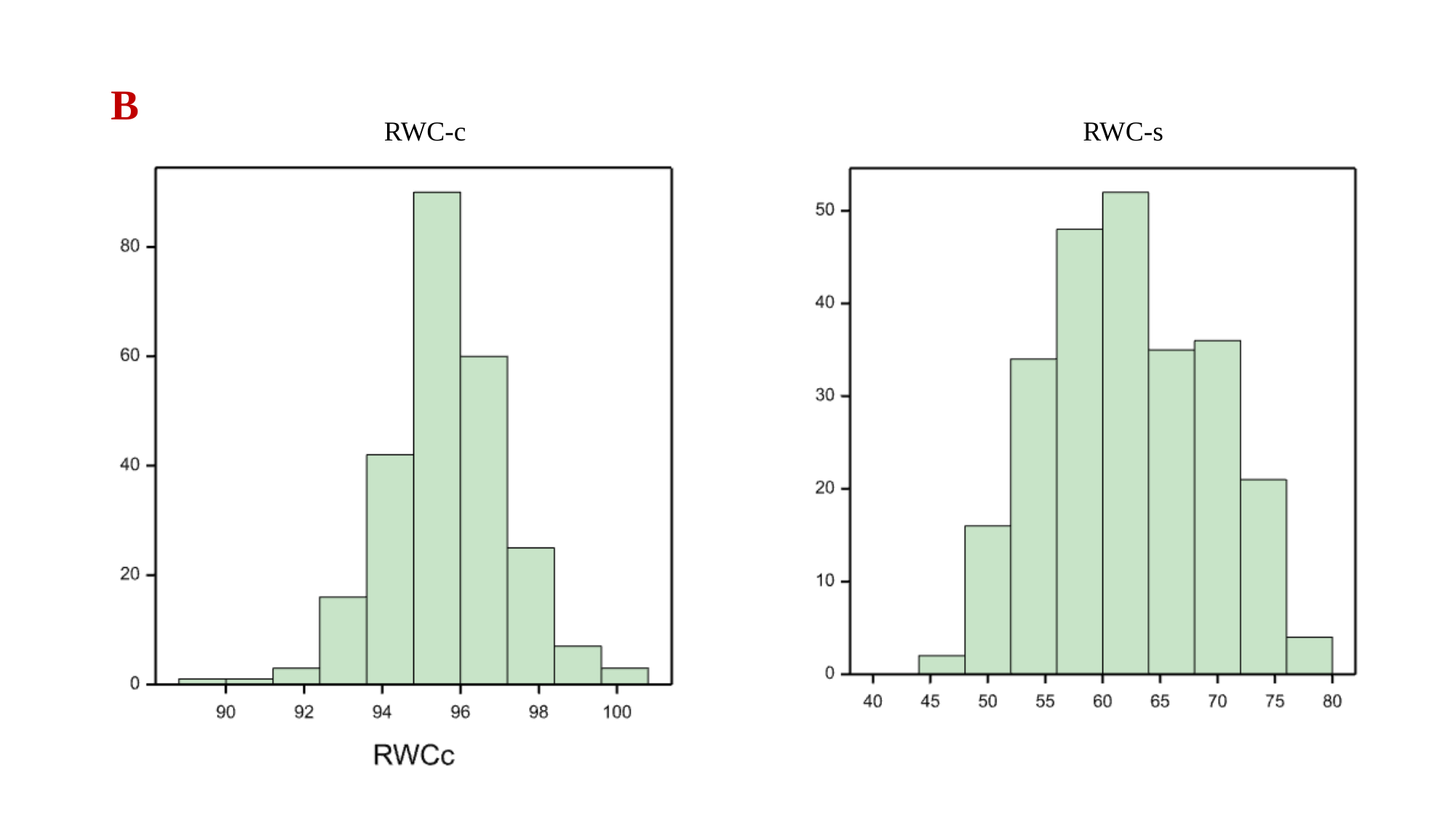

B
RWC-c
RWC-s

## Slide 3
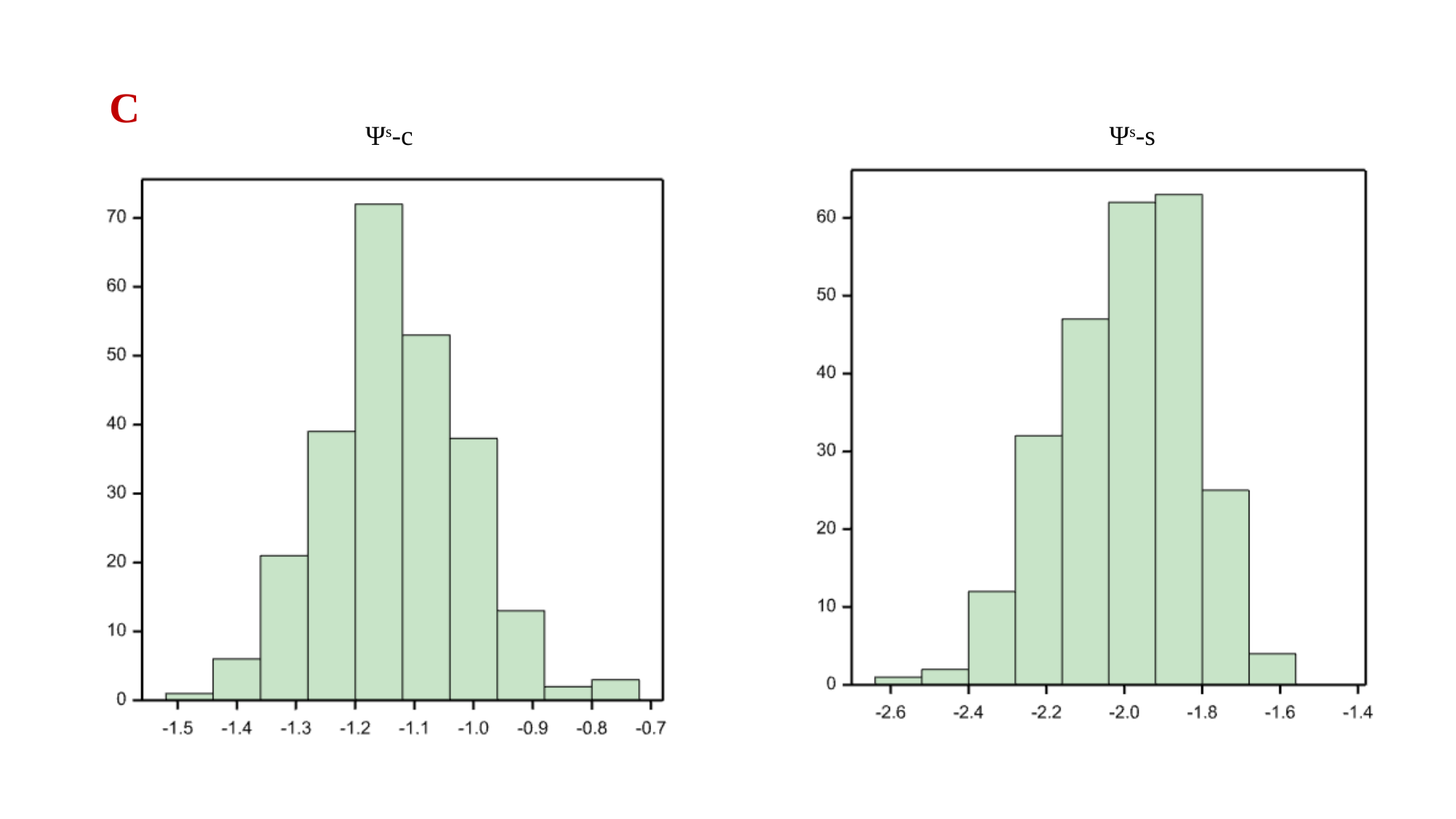

C
Ψs-c
Ψs-s

## Slide 4
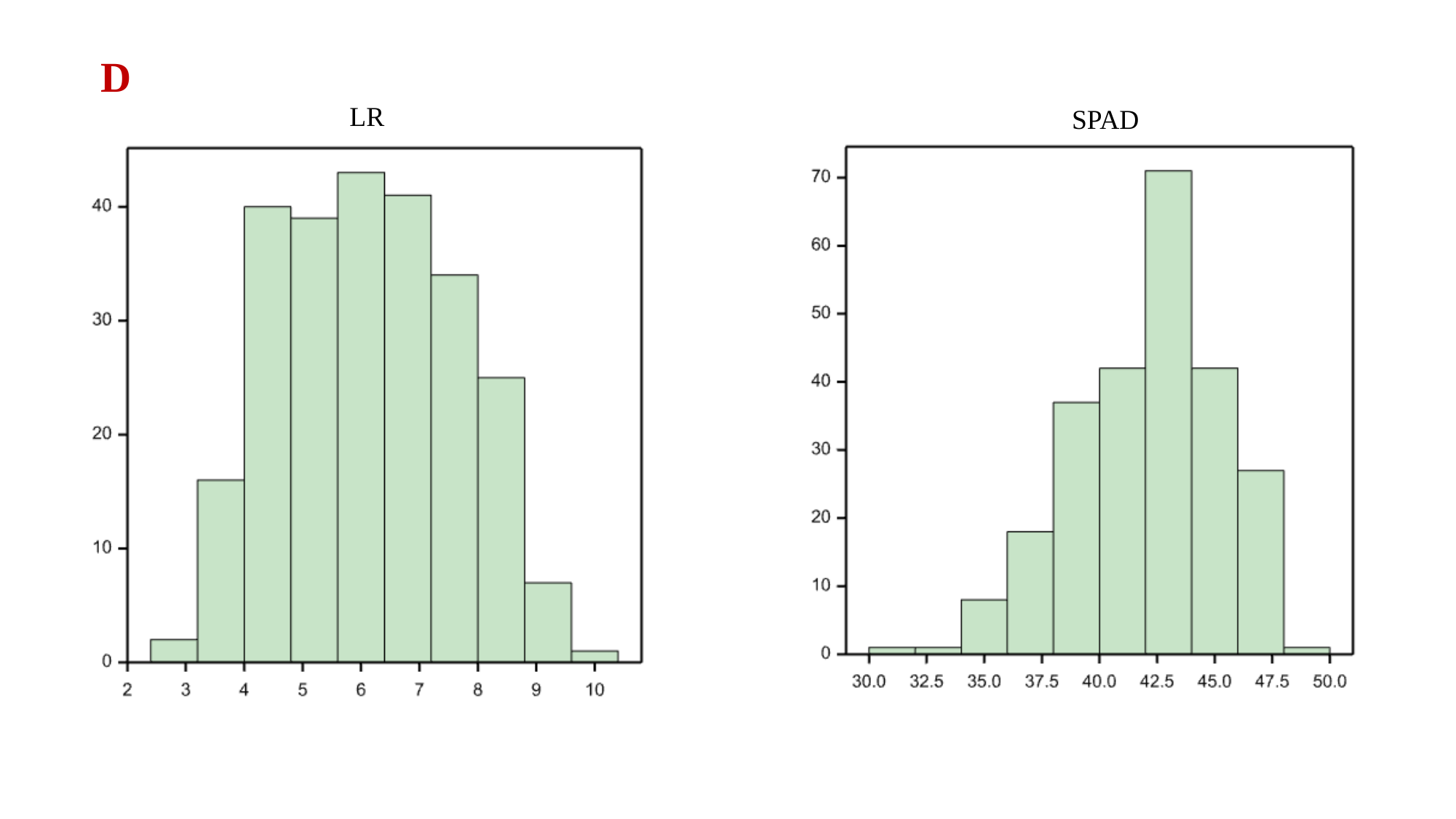

D
LR
SPAD
