# Supplementary material for: Genome Wide Association Study Uncovers the QTLome for Osmotic Adjustment and Related Drought Adaptive Traits in Durum Wheat
Source: Genes (Basel). 2022 Feb 2;13(2):293. doi: 10.3390/genes13020293 (PMC8871942; doi:10.3390/genes13020293)
Supplement: Supplementary file 1 [file genes-13-00293-s001.zip › Supplementary material final/Supplementary material GEC_24.1.2022 2/Table S4.pdf]

Table S4. List of Flowering Time (FT) QTLs.

Flowering Time (FT)							
QTL	Marker	Chr.	Position (cM)	Log P value	R^2	Allele	Effect
QFT.ubo-1A.1	IWA3089	1A	142.1	4.34	5.39	A/G	6.17
QFT.ubo-2A.1	IWB62185	2A	24.7	5.85	7.65	C/T	-12.40
QFT.ubo-2A.2	IWA2526	2A	46.6	5.30	6.81	C/T	-6.68
QFT.ubo-2A.3	IWB26001	2A	198.7	5.01	6.38	A/G	16.50
QFT.ubo-2B.1	IWB32315	2B	5.9	4.06	4.98	A/G	-6.40
QFT.ubo-4A.1	IWB13323	4A	23.7	5.39	6.94	C/T	-12.46
QFT.ubo-4A.2	IWB43659	4A	107.1	4.10	5.03	A/G	-5.08
QFT.ubo-4A.3	IWB42413	4A	168.5	4.02	4.91	A/G	5.49
QFT.ubo-4B.1	IWA2125	4B	15.5	3.10	3.60	C/T	-6.28
QFT.ubo-5A.1	IWB35931	5A	43.8	3.27	3.85	C/G	-9.09
QFT.ubo-5A.2	IWB12396	5A	68.2	4.70	5.91	C/T	5.48
QFT.ubo-5A.3	IWB14493	5A	95.6	3.04	3.52	C/T	4.61
QFT.ubo-6A.1	IWB53923	6A	49.5	5.05	6.44	C/T	-10.86
QFT.ubo-6B.1	IWB14571	6B	71.9	5.36	6.90	A/G	-9.23
QFT.ubo-7B.1	IWB371	7B	177.1	4.26	5.27	A/G	6.36
